# Phagocytosis-inducing antibodies to *Plasmodium falciparum* upon immunization with a recombinant PfEMP1 NTS-DBL1α domain

**DOI:** 10.1186/s12936-016-1459-3

**Published:** 2016-08-17

**Authors:** Maria del Pilar Quintana, Davide Angeletti, Kirsten Moll, Qijun Chen, Mats Wahlgren

**Affiliations:** 1 Department of Microbiology, Tumor and Cell Biology (MTC), Karolinska Institutet, Stockholm, Sweden; 2 Escuela de Medicina y Ciencias de la Salud, Facultad de Ciencias Naturales y Matemáticas, Universidad del Rosario, Bogotá, Colombia; 3 Key Laboratory of Zoonosis, Jilin University, Changchun, People’s Republic of China; 4 Institute of Pathogen Biology, Chinese Academy of Medical Sciences, Beijing, People’s Republic of China

**Keywords:** Opsonization, Phagocytosis, Rosetting, Antibodies, PfEMP1, NTS-DBL1α

## Abstract

**Background:**

Individuals living in endemic areas gradually acquire natural immunity to clinical malaria, largely dependent on antibodies against parasite antigens. There are many studies indicating that the variant antigen PfEMP1 at the surface of the parasitized red blood cell (pRBC) is one of the major targets of the immune response. It is believed that antibodies against PfEMP1 confer protection by blocking sequestration (rosetting and cytoadherence), inducing antibody-dependent cellular-inhibitory effect and opsonizing pRBCs for phagocytosis.

**Methods:**

A recombinant NTS-DBL1α domain from a rosette-mediating PfEMP1 was expressed in *Escherichia coli.* The resulting protein was purified and used for immunization to generate polyclonal (goat) and monoclonal (mouse) antibodies. The antibodies’ ability to opsonize and induce phagocytosis in vitro was tested and contrasted with the presence of opsonizing antibodies naturally acquired during *Plasmodium falciparum* infection.

**Results:**

All antibodies recognized the recombinant antigen and the surface of live pRBCs, however, their capacity to opsonize the pRBCs for phagocytosis varied. The monoclonal antibodies isotyped as IgG2b did not induce phagocytosis, while those isotyped as IgG2a were in general very effective, inducing phagocytosis with similar levels as those naturally acquired during *P. falciparum* infection. These monoclonal antibodies displayed different patterns, some of them showing a concentration-dependent activity while others showed a prozone-like effect. The goat polyclonal antibodies were not able to induce phagocytosis.

**Conclusion:**

Immunization with an NTS-DBL1-α domain of PfEMP1 generates antibodies that not only have a biological role in rosette disruption but also effectively induce opsonization for phagocytosis of pRBCs with similar activity to naturally acquired antibodies from immune individuals living in a malaria endemic area. Some of the antibodies with high opsonizing activity were not able to disrupt rosettes, indicating that epitopes of the NTS-DBL1-α other than those involved in rosetting are exposed on the pRBC surface and are able to induce functional antibodies. The ability to induce phagocytosis largely depended on the antibody isotype and on the ability to recognize the surface of the pRBC regardless of the rosette-disrupting capacity.

**Electronic supplementary material:**

The online version of this article (doi:10.1186/s12936-016-1459-3) contains supplementary material, which is available to authorized users.

## Background

Malaria is a devastating parasitic disease endemic in over 97 countries, representing around 3.3 billion people at risk of suffering the disease. There are around 149–303 million malaria cases per year and an estimated of 438,000 deaths, most of which are attributed to *Plasmodium falciparum* infections [1]. The majority of the malaria clinical symptoms are associated with the parasite’s asexual cycle inside the host’s red blood cells (RBCs). This intra-erythrocytic cycle involves extensive modification of the host cell, through the transport of parasite-derived proteins to the RBC cytoplasm and plasma membrane. The major surface antigen *P. falciparum* erythrocyte membrane protein 1 (PfEMP1) [2] belongs to a large multi-domain protein family (between 200 and 350 kDa), encoded by the hypervariable *var* gene family [2–4]. *var* genes are between 6 and 14 kb and have two exons separated by a conserved intron. The first exon encodes a hypervariable extracellular binding region, which includes an N-terminal segment (NTS) and multiple adhesive domains of duffy binding-like (DBL)-type or cysteine-rich interdomain region (CIDR)-type, sometimes interspersed with C2 inter-domains. The second exon encodes a transmembrane (TM) segment and a more conserved acidic terminal segment (ATS). The DBL and CIDR domains are numbered consecutively from the N-terminus and have been respectively classified into six (α, β, γ, δ, ε and ζ) and five (α, β, γ, δ and pam) different types based on sequence similarities [5, 6]. PfEMP1 is used by the parasite to evade clearance from the human host through two main mechanisms: (1) evasion of the host immune response raised against one PfEMP1 variant, switching to new variants through differential expression of approximately 60 distinct members per genome of the* var* family [3]; and, (2) evasion of the spleen clearance through sequestration of the pRBCs in the host’s microvasculature [7, 8], which is mediated by the interaction between PfEMP1 and receptors located on the endothelial cell surface (cytoadhesion) or receptors on the uninfected RBC surface (rosetting). Among the different DBL domains, the DBL1α is the most conserved [9, 10] and has been identified as a ligand both for rosetting and cytoadhesion [11–13].

After prolonged exposure, individuals living in endemic areas develop immunity to malaria, which manifests as an age-associated decline in the prevalence of severe, then mild clinical episodes [14]. This naturally acquired immunity to malaria is in part due to antibodies since passive transfer of sera from clinically immune adults to children infected with malaria decreases the parasitaemia and reduces the clinical symptoms [15]. There are a large number of studies indicating that the variant antigen PfEMP1 at the surface of the pRBC is the major target of the variant-specific immune response [16–20], and the antibodies are believed to confer protection by blocking sequestration, inducing antibody-dependent cellular-inhibition (ADCI) and opsonizing the pRBCs for phagocytosis. However, the actual variants and epitopes involved as well as the composition, specificity and importance of each possible protective mechanism underlying the naturally acquired anti-PfEMP1 immunity are still unclear. The first antibody protective mechanism has been extensively studied, showing that antibodies against the NTS-DBL1α-domain both disrupt rosettes in vitro and protect against the sequestration of pRBC in vivo [21, 22], however it is unclear whether other protective and less studied roles (e.g., pRBC opsonization for phagocytosis) act synergistically with the anti-rosetting/cytoadhesion activity as a possible combined mechanism for parasite clearance. An in vitro study suggests that this is the case [22].

To explore this possibility a recombinant NTS-DBL1α-domain from a rosette-mediating PfEMP1 variant was expressed, in order to investigate if the antibodies elicited upon animal immunization with this domain were able to inhibit rosetting and more importantly to opsonize and induce pRBC phagocytosis in vitro.

## Methods

### Parasite cultures

*Plasmodium falciparum* FCR3S1.2 parasites were cultured according to standard methods, and the rosetting phenotype was maintained by enrichment over a Ficoll-gradient [23].

### Production of recombinant protein

Construct expression of the NTS-DBL1α-domain was performed as previously described [24]. In brief, the NTS-DBL1α-domain spanning amino acids 1–481 (PFIT_bin06900) was codon optimized for expression in bacteria and cloned into the pJ414express vector (DNA2.0). The protein was expressed as a C-terminal 6× histidine-tagged recombinant protein in *Escherichia coli* (Shuffle T7, New England Biolabs); bacteria were grown at 30 °C until they reached an OD_600_ = 0.6–0.8 and then induced with 0.4 mM IPTG (isopropyl-β-D-thiogalactopyranoside) for 20 h at 16 °C. Pelleted bacteria were lysed by sonication and the recombinant protein was recovered from the soluble fraction after centrifugation. The protein was purified by immobilized metal affinity chromatography (IMAC) over a TALON Cobalt column (Clontech), followed by size exclusion chromatography on a HiLoad 16/60 Superdex 75 pg column (GE-Healthcare). The purified protein was analysed by sodium dodecyl sulfate polyacrylamide gel electrophoresis (SDS-PAGE) and Western blot using an antibody against the poly-His tag (Qiagen).

### Generation of monoclonal and polyclonal antibodies

Monoclonal and polyclonal antibodies against the recombinant NTS-DBL1α domain were produced as previously described [24, 25]. Nine mouse monoclonal antibodies were produced in collaboration with the EMBL Monoclonal Antibody Core Facility, Monterotondo, Italy. Briefly, mice were immunized three times with 50 μg of recombinant protein. Antibody levels were measured prior and post fusion by ELISA to select positive cell clones. Monoclonal antibodies were purified over a Protein G agarose column (Pierce ThermoScientific) and subsequently dialyzed and concentrated.

Goat polyclonal antibodies were produced by Agrisera (Vännäs, Sweden). One goat was immunized four times at one-month intervals with 200 μg of protein emulsified in Freund’s complete for the first immunization and incomplete adjuvant for the following three immunizations. Final bleeding was carried out 2 weeks after the last immunization and the total IgG was purified on Protein G columns. A set of six human sera from Myanmarese immune adults was used to test for the presence of naturally acquired pRBCs-opsonizing antibodies, able to induce phagocytosis by THP-1 cells in vitro. Sera were collected from asymptomatic adults (18–40 years of age and tested positive for *P. falciparum* infection by microscopy) residents of a malaria endemic area in northern Myanmar (Kachin province).

### Enzyme-linked immunosorbent assay (ELISA)

For the immune sera set, antibodies against the recombinant NTS-DBL1α were measured by ELISA as previously described [26]. Maxisorp plates (Nunc, Rosklid) were coated overnight at 4 °C with 1 μg of the recombinant antigen dissolved in 15 mM Na_2_CO_3_ and 35 mM NaHCO_3_ (pH 9.6). Plates were washed three times with PBS containing 0.1 % Tween 20 (PBST) and then blocked with 1 % bovine serum albumin (BSA) in PBST for 1 h at room temperature and subsequently washed three times with PBST. All samples were titrated by six serial dilutions in PBS to determine concentration dependency, and they were incubated in triplicate for 1 h at room temperature. Plates were washed three times with PBST, then bound IgG was measured by incubation for 1 h at room temperature with alkaline phosphatase-conjugated goat anti-human IgG (Sigma) diluted 1:1000 in PBS. Plates were washed three times with PBST and developed with SigmaFast p-nitrophenyl phosphate tablets (Sigma) for 15 min. The optical density (OD) was measured at 405 nm in an ELISA reader (Multiskan™ GO Microplate Spetrophotometer, Thermo Scientific). A control pool of six non-malaria exposed Swedish donors was included, as well as control wells without serum (background). Seropositivity was determined based on the 1:500 dilutions as the mean OD_405_ plus two standard deviations of the Swedish control pool.

### Flow cytometry assay on infected erythrocytes

Antibody binding to pRBCs was tested using flow cytometry as previously described [27]. Briefly, the pRBCs were blocked for 1 h with 2 % fetal bovine serum (FBS) in PBS followed by incubation with the human serum samples in a dilution 1:5 for 30 min at room temperature. The pRBCs were washed three times with 2 % FBS in PBS followed by incubation for 30 min at room temperature and protected from light with a goat anti-human IgG antibody coupled to Alexa488 (Molecular Probes®, Life Technologies, dilution 1:200) and ethidium bromide at a final concentration of 2.5 μg/ml to stain the parasite nuclei (added during the last 10 min of the incubation). Finally the pRBCs were washed three times with 2 % FBS in PBS followed by flow cytometry analysis. A control pool of six non-malaria exposed Swedish donors was included. The surface reactivity was expressed as geometric mean fluorescent intensity (MFI) and adjusted according to non-malaria exposed control reactivity and the non-parasitized RBCs reactivity according to the following formula: (pRBCimmune/pRBCnon-malaria)−(RBCimmune/RBCnon-malaria). Samples were considered positive when the adjusted MFI was higher than zero as reported previously [28].

### Rosette disruption assay

The ability of human serum to disrupt rosettes was tested as described before [29]. A control pool of six non-malaria exposed Swedish donors was included as well as a negative control where no serum was added. Each serum sample was tested in a 1:5 dilution in 50 μl of parasite suspension, at 5 % haematocrit and at least 5 % parasitaemia. The samples were incubated in duplicates at room temperature for 1 h, followed by parasite staining with acridine orange and counting of rosettes under the microscope. For each sample at least 200 pRBCs were counted and the rosetting rate in the presence of serum was calculated relative to the rosetting rate in the negative control (no serum added). Samples were considered positive if disruption was >15 % compared to the control.

The presence of antibodies against the recombinant NTS-DBL1α, the recognition of the native homologous protein expressed on the surface of pRBCs and the rosette disruption activity for the monoclonal and the polyclonal antibodies were measured using similar methodologies and the results were published elsewhere [25].

### Phagocytosis assay

The phagocytosis assay was performed as described before [22] with modifications. The human monocytic line THP-1 was maintained in RPMI 1640 supplemented with 2 mM glutamine, 0.85 g/L NaHCO_3_ (Thermo Scientific) 10 % inactivated fetal bovine serum (FBS), 1000 U/ml of penicillin, 1 mg/ml of streptomycin (Sigma) and 20 mM HEPES at a maximum density of 1 × 10^6^ cells/ml. The cells were checked periodically for surface expression of the Fcγ-receptors I (CD64) and II (CD32) on the surface, which is necessary for the antibody-dependent phagocytic activity [30, 31]. In brief THP-1 cells were separately stained with anti-CD32, anti-CD64 or anti-CD16 antibodies (FITC mouse anti-human, 552,883, 555,527 and 556,618, BD Biosciences, 1:10 dilution) and checked by flow cytometry.

Highly synchronized FCR3S1.2 pRBCs (30–32 hpi, 10 % parasitaemia and >70 % rosetting) were used for the phagocytosis assays. They were washed in malaria culture medium (MCM: RPMI 1640, 20 mM HEPES, 0.85 g/L NaHCO_3_, 2 mM glutamine, 25 μg/ml Gentamicin); the rosettes were disrupted mechanically (using a 23G syringe) and the pRBCs purified using a VarioMACS magnet. The final parasitaemia and the total cell number were determined and only samples with >80 % purity were used. The pRBCs were resuspended in 2.5 μg/ml ethidium bromide solution in MCM, adjusting the pRBCs’ density to 3.3 × 10^7^ cells/ml. From this suspension, 30 μl per well were distributed in a rounded-bottomed, 96-well plate, the cells were posteriorly pelleted down and re-suspended in the appropriate antibody concentration solution in MCM. A positive (rabbit anti human red blood cells, ab34858, ABCAM, 1:100 dilution) and a negative control (unopsonized control) were always included. Other controls were included: a monoclonal antibody against a non-malaria related antigen (the bacterial protein SlyD), non-immune goat IgG and non-immune Swedish sera controls. The opsonization was performed at 37 °C for 45 min, followed by three washes with MCM and re-suspension in THP-1 cells culture medium. THP-1 cells were washed once and re-suspended at an adjusted density of 5 × 10^5^ cells/ml; 100 μl of this suspension were dispensed in a separate rounded-bottomed, 96-well plate, 50 μl of the opsonized pRBCs were dispensed onto the THP-1 cells mixed gently and incubated for 40 min at 37 °C, 5 % CO_2_. The phagocytosis was stopped by centrifugation at 4 °C followed by re-suspension in room temperature ammonium chloride lysing solution (15 mM NH_4_Cl, 10 mM NaHCO_3_, 1 mM EDTA) for 3 min to lyse non-ingested pRBCs. The lysis was stopped by addition of PBS supplemented with 2 % FBS, followed by three washes. After the final wash the cells were analysed by flow cytometry (FACSCalibur, BD Biosciences), gating for THP-1 cells and determining the percentage of ethidium bromide positive cells (FL2 positive cells). The phagocytosis rate was calculated relative to the percentage of ethidium bromide positive cells in the positive control and antibodies were considered as positive if relative phagocytosis was higher than the negative control (mAb SlyD for the monoclonals, control goat IgG for the ITvar60 goat IgG and Swedish control pool for the human serum samples) plus two standard deviations.

### Analysis

Flow cytometry analysis was performed using the FlowJo version 9.2 software (TreeStar, USA). Mean, standard deviations (SD) and figures were performed using the GraphPad Prism version 6.0f for Mac OS X (La Jolla, CA, USA). All values are expressed as mean ± SD from three independent experiments.

## Results

The recombinant NTS-DBL1α-domain from the rosette-mediating PfEMP1 IT4var60 (PFIT_bin06900) was expressed as a C-terminal His-tagged protein in *E. coli*. The protein migrated as a monomer under non-reducing SDS-PAGE with the expected molecular weight of 55 kDa, and it was also detected by an anti-His on Western blot experiments.

**Fig. 1:**
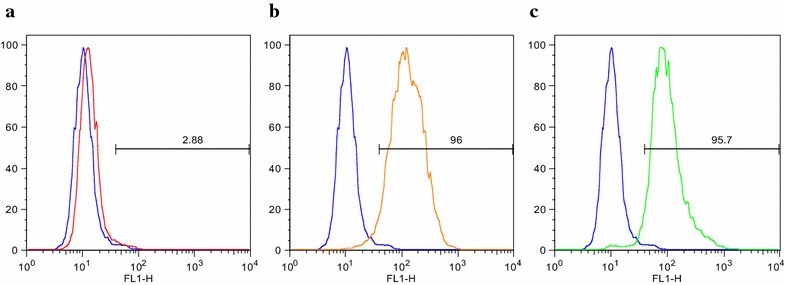
Fcγ receptors on the THP-1 cell surface. THP-1 cells were stained with **a** FITC anti-CD16 (*red*), **b** anti-CD32 (*orange*) and **c** anti-CD64 (*green*) antibodies and visualized by flow cytometry. Unstained cells are shown in *blue*

The human monocytic cell line THP-1 was used to perform the phagocytosis assays due to its well-documented phagocytic activity against opsonized particles (e.g., latex beads and RBCs) and apoptotic bodies [32]. Since the antibody-dependent phagocytic activity requires the presence of Fc receptors on the surface of the phagocytic cell, the THP-1 line was periodically checked for surface expression of the Fcγ-receptors I (CD64) and II (CD32). Flow cytometry showed the expected positive expression of CD32 and CD64 in more than 90 % of the cells while CD16 was negative as previously reported for this cell line (Fig. 1) [30, 31].

**Fig. 2:**
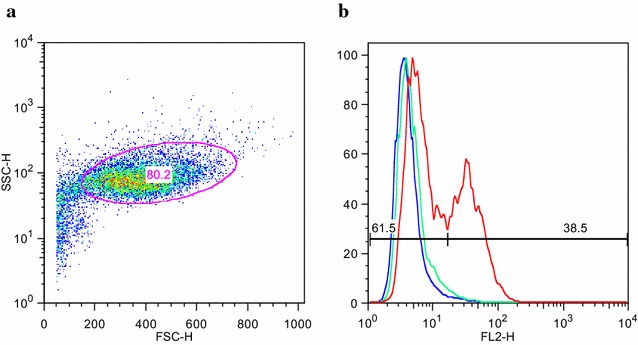
Flow cytometry analysis of THP-1 cells after incubation with antibody-opsonized pRBCs. **a** THP-1 cells were gated on *light scattered* characteristics and **b** the percentage of ethidium bromide positive cells was calculated. THP-1 cells alone are depicted in *blue*, THP-1 cells incubated with unopsonized pRBCs in *green* and THP-1 cells incubated with pRBCs opsonized with a positive control in *red*

To investigate whether antibodies elicited upon animal immunization with the recombinant NTS-DBL1α-domain (from a rosette-mediating PfEMP1 variant) and antibodies present in adult immune sera from a malaria-endemic area were able to opsonize and induce pRBC phagocytosis in vitro, fluorescently labelled FCR3S1.2 pRBCs were pre-incubated with the antibodies and then mixed with THP-1 cells. In order to determine the proportion of THP-1 cells that had phagocytosed labelled pRBCs, THP-1 cells were identified based on light scatter characteristics and the percentage of ethidium bromide positive cells determined (Fig. 2).

**Fig. 3:**
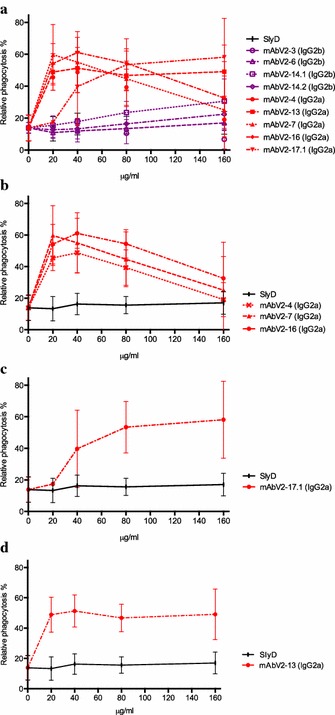
Phagocytosis of FCR3S1.2 pRBCs after opsonization with monoclonal anti-PfEMP1 antibodies. Antibodies were tested in serial dilutions between 20 and 160 μg/ml. Relative phagocytosis was calculated as the percentage of ethidium bromide THP-1 positive cells relative to the positive control (rabbit anti human red blood cells, ab34858, ABCAM, 1:100 dilution). The negative control (unopsonized) was media alone (no antibody added). Values shown are mean and standard deviation from triplicates. **a** Antibodies isotyped as IgG2b are depicted in *purple* and those isotyped as IgG2a are depicted in *red*. **b** Mouse monoclonal antibodies showing a prozone-like effect with phagocytosis increasing with initial increases in antibody concentration to then decrease when higher antibody concentrations were used. **c** A single mouse monoclonal antibody showing gradual increases in phagocytosis with increasing antibody concentrations. **d** A single mouse monoclonal antibody showing high and stable phagocytosis induction at all antibody concentrations, being the most effective even at low concentrations

The mouse monoclonal antibodies showed variable abilities to opsonize and induce phagocytosis. In general, all antibodies isotyped as IgG2b were not able to induce phagocytosis (Fig. 3a), generating values similar to those of the negative control (unopsonized) and of the non-malaria-related mouse monoclonal antibody (mAbSlyD). This can be explained by the poor binding of this particular isotype to human Fcγ-RI (CD64) [33]. Conversely, antibodies isotyped as IgG2a were able to induce various magnitudes and patterns of phagocytosis (Fig. 3a). The different patterns of phagocytosis were categorized as follows: (1) a prozone-like behaviour, with phagocytosis increasing with initial increases in antibody concentration (up to 40 μg/ml) followed by a decrease when higher antibody concentrations were used (mAbV2–4, mAbV2–7 and mAbV2–16, Fig. 3b); (2) a gradual increase in phagocytosis with increasing antibody concentrations (mAbV2–17.1, Fig. 3c); and, (3) a high and stable phagocytosis induction at all antibody concentrations (mAbV2–13, Fig. 3d). When comparing the monoclonal antibodies in rosette disruption and opsonization for phagocytosis (Table 1), three groups of surface-reacting antibodies emerged: (1) a group effective in both assays (mAbV2–7, mAbV2–13 and mAbV2–17.1); (2) a group effective in rosette disruption but not in opsonization for phagocytosis (mAbV2–3, mAbV2–6, mAbV2–14.1 and mAbV2–14.2), where each group’s ability to induce phagocytosis is linked to their isotype (IgG2a vs IgG2b); and, (3) a group effective in opsonization for phagocytosis but not in rosette disruption (mAbV2–4 and mAbV2–16), indicating that other regions of the NTS-DBL1-α besides those involved in rosetting are exposed on the pRBC surface and are able to induce antibodies with effector functions. A previous study has described a surface-exposed epitope in the sub-domain 2 (SD2) of the DBL-1α that is able to induce strain-transcending and surface-reacting antibodies that do not disrupt rosettes [28]. The data presented here contrast with a previous study that describes a clear positive correlation between rosette disruption and opsonization for phagocytosis [22]. Instead, the data showed that the most important factors defining the level of phagocytosis are antibody surface reactivity and isotype, independently of the ability to disrupt rosettes.

**Table 1:** Properties of the antibodies used in the study

	Antibody type	ELISA-reactivity (ITvar60 NTS-DBL1α)	Surface reactivity (FCR3S1.2)	Rosette disruption (FCR3S1.2)	Opsonization for phagocytosis (FCR3S1.2)
mAbV2–3 (IgG2b)^a^	IgG2b	+	+	+	−
mAbV2–6 (IgG2b)^a^	IgG2b	+	+	+	−
mAbV2–14.1 (IgG2b)^a^	IgG2b	+	+	+	−
mAbV2–14.2 (IgG2b)^a^	IgG2b	+	+	+	−
mAbV2–4 (IgG2a)^a^	IgG2a	+	+	−	+
mAbV2–7 (IgG2a)^a^	IgG2a	+	+	+	+
mAbV2–13 (IgG2a)^a^	IgG2a	+	+	+	+
mAbV2–16 (IgG2a)^a^	IgG2a	+	+	−	+
mAbV2–17.1 (IgG2a)^a^	IgG2a	+	+	+	+
mAbSly D^a^	IgG2b	−	−	−	−
ITvar60 goat IgG^a^	IgG	+	+	+	−
Control goat IgG^a^	IgG	−	−	−	−
IM1	Serum	+	+	−	+
IM2	Serum	+	+	−	+
IM3	Serum	+	+	−	+
IM4	Serum	+	+	−	+
IM5	Serum	+	+	−	+
IM6	Serum	+	+	+	+
IMP	Serum	+	+	−	+
SCP	Serum	−	−	−	−

Table summarizes the sample name (*mAb* monoclonal antibody, *IM* immune malaria, *IMP* immune malaria pool, *SCP* Swedish control pool) and antibody type with their corresponding activity (±) in different assays. Positivity was determined based on the cut-off criteria specified in the “Methods” section

^a^ELISA reactivity, surface reactivity and rosette disruption activity for these antibodies have been published elsewhere [25] and are accordingly reported here

**Fig. 4:**
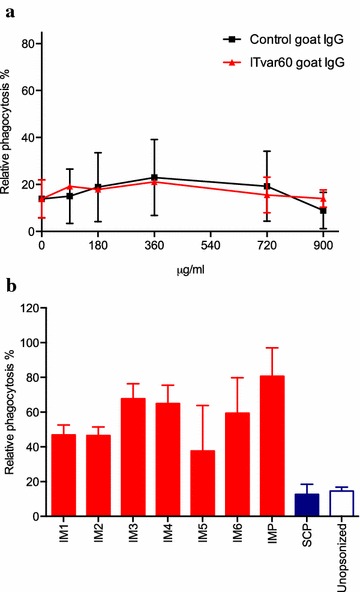
Phagocytosis of FCR3S1.2 pRBCs after opsonization with polyclonal anti-PfEMP1 antibodies or sera from human immune samples. **a** Capacity of the ITvar60goat IgG to opsonize and induce phagocytosis of FCR3S1.2 pRBCs. Antibodies were tested in serial dilutions between 90 and 900 μg/ml. **b** Capacity of the immune sera (IM1–IM6 and pooled together IMP) to opsonize and induce phagocytosis of FCR3S1.2 pRBCs. All sera samples were tested at 1:5 dilution. Pooled Swedish non-immune control is depicted in *blue*

Polyclonal antibodies generated in goat were able to recognize the recombinant antigen (measured by ELISA) as well as the native protein expressed on the surface of pRBCs (measured by flow cytometry); furthermore, they disrupted the rosettes (Table 1). However, they were not able to induce phagocytosis by the THP-1 cells in vitro. High antibody concentrations (900 μg/ml) generated phagocytosis values similar to total IgG purified from a control goat (Fig. 4a) and attempts to use lower concentrations (<10 μg/ml) to rule out a possible prozone-like effect were unsuccessful. In goats, two IgG isotypes are present: IgG1 and IgG2; only IgG1 is peaking in concentration during immune responses while IgG2, to which cytophilic activity is attributed, remains stable. However, human Fc receptors seem to have a very low affinity for goat IgG [34, 35] explaining the absence of phagocytosis induction by the polyclonal IgG used in this study.

The set of six human sera from Myanmarese immune adults (individually or pooled together) showed the presence of potent, naturally acquired, pRBCs-opsonizing antibodies able to induce phagocytosis (Fig. 4b) as has been shown in other studies [36, 37]. However, even though antibodies against the particular NTS-DBL1-α variant expressed by the parasite used (measured by ELISA Additional file 1: Figure S2) were detected in all samples, the observed reactivity against the pRBCs surface (Additional file 2: Figure S1) and the induction of phagocytosis may also originate from other surface-directed antibodies. It is likely that antibodies recognizing other surface-exposed antigens, e.g., other PfEMP1 domains, RIFINs, SURFINs, STEVORs, and possibly others, are present in the samples. Previous studies have suggested that anti-RIFIN antibodies are a dominant component of the overall response against surface-exposed antigens [38, 39], and the variability confined in the predicted extracellular segment of SURFIN_4.2_ (the member of the protein family transported to the surface) suggests that it is also likely to be exposed to the immune system and to be a target of positive selection [40]. Therefore, it is still important to convincingly identify if there are additional targets, besides PfEMP1, among the host’s naturally acquired, opsonizing antibodies, particularly in African children who are at the greatest risk of malaria. A recent study has shown correlation between protection against clinical malaria and naturally acquired, opsonizing antibodies against *P. falciparum* merozoites [41]; it would be interesting to investigate the same protective correlation with opsonizing antibodies against pRBCs.

## Conclusion

In summary, the results showed that immunization with a PfEMP1 NTS-DBL1-α domain elicits antibodies that not only have a biological role in rosette disruption, and therefore in sequestration blocking, but also effectively induce opsonization for phagocytosis of pRBCs, a role that could be of great importance during pRBCs clearance in vivo. Moreover, some of the antibodies with high opsonizing activity were not able to disrupt rosettes, indicating that epitopes of the NTS-DBL1-α other than those involved in rosetting are exposed on the pRBC surface and are able to induce functional antibodies that could provide protection.

## Additional files

10.1186/s12936-016-1459-3 IgG levels in the human samples against the recombinant NTS-DBL1α (ITvar60) measured by ELISA. The original OD values were fitted to a 4 Parameter Logistic (4PL) curve. The antibody concentration is expressed as log of the dilution factor (DF). Immune samples are depicted in red and named from 1 to 6, The pooled immune sample (IMP) is also depicted. Swedish control pool (SCP) is depicted in blue.
10.1186/s12936-016-1459-3 Surface reactivity of the human samples measured by FACS. Immune samples are depicted in red and named from 1 to 6, The pooled immune sample (IMP) is also depicted. Swedish control pool (SCP) is depicted in blue.

## Abbreviations

NTS-DBL1-α: N-terminal segment-Duffy-binding like alpha
PfEMP1: *Plasmodium falciparum* erythrocyte membrane protein 1
pRBC: parasitized red blood cell
ADCI: antibody-dependent cellular-inhibitory effect
CIDR: cysteine-rich interdomain region
TM: transmembrane
ATS: acidic terminal segment

## References

[1] WHO (2015). World malaria report 2015.

[2] Smith JD, Chitnis CE, Craig AG, Roberts DJ, Hudson-Taylor DE, Peterson DS (1995). Switches in expression of *Plasmodium falciparum* VW genes correlate with changes in antigenic and cytoadherent phenotypes of infected erythrocytes. Cell.

[3] Su XZ, Heatwole VM, Wertheimer SP, Guinet F, Herrfeldt JA, Peterson DS (1995). The large diverse gene family var encodes proteins involved in cytoadherence and antigenic variation of *Plasmodium falciparum*-infected erythrocytes. Cell.

[4] Baruch DI, Pasloske BL, Singh HB, Bi X, Ma XC, Feldman M (1995). Cloning the *P. falciparum* gene encoding PfEMPl, a malarial variant antigen and adherence receptor on the surface of parasitized human erythrocytes. Cell.

[5] Smith JD, Subramanian G, Gamain B, Baruch DI, Miller LH (2000). Classification of adhesive domains in the *Plasmodium falciparum* erythrocyte membrane protein 1 family. Mol Biochem Parasitol.

[6] Rask TS, Hansen DA, Theander TG (2010). Gorm Pedersen A, Lavstsen T. *Plasmodium falciparum* erythrocyte membrane protein 1 diversity in seven genomes—divide and Conquer. PLoS Comput Biol.

[7] Turner GDH, Morrison H, Jones M, Davis TME, Looareesuwan S, Buley ID (1994). An immunohistochemical study of the pathology of fatal malaria evidence for widespread endothelial activation and a potential role for intercellular adhesion molecule-1 in cerebral sequestration. Am J Pathol.

[8] Silamut K, Phu NH, Whitty C, Turner GD, Louwrier K, Mai NT (1999). A quantitative analysis of the microvascular sequestration of malaria parasites in the human brain. Am J Pathol.

[9] Flick K, Chen Q (2004). var genes, PfEMP1 and the human host. Mol Biochem Parasitol.

[10] Kraemer SM, Kyes SA, Aggarwal G, Springer AL, Nelson SO, Christodoulou Z (2007). Patterns of gene recombination shape var gene repertoires in *Plasmodium falciparum*: comparisons of geographically diverse isolates. BMC Genom.

[11] Rowe JA, Moulds JM, Newbold CI, Miller LH (1997). *P. falciparum* rosetting mediated by a parasite-variant erythrocyte membrane protein and complement-receptor 1. Nature.

[12] Chen Q, Barragan A, Fernandez V, Sundström A, Schlichtherle M, Sahlén A (1998). Identification of *Plasmodium falciparum* erythrocyte membrane protein 1 (PfEMP1) as the rosetting ligand of the malaria parasite *P. falciparum*. J Exp Med.

[13] Chen Q, Heddini A, Barragan A, Fernandez V, Pearce SFA, Wahlgren M (2000). The semiconserved head structure of *Plasmodium falciparum* erythrocyte membrane protein 1 mediates binding to multiple independent host receptors. J Exp Med.

[14] Trape J-F, Rogier C, Konate L, Diagne N, Bouganali H, Canque B (1994). The Dielmo project: a longitudinal study of natural malaria infection and the mechanics of protective immunity in a community living in a holoendemic area of Senegal. Am J Trop Med Hyg.

[15] Cohen S, McGregor IA, Carrington S (1961). Gamma-globulin and acquired immunity to human malaria. Nature.

[16] Marsh K, Otoo L, Hayes RJ, Carson DC, Greenwood BM (1989). Antibodies to blood stage antigens of *Plasmodium falciparum* in rural Gambians and their relation to protection against infection. Trans R Soc Trop Med Hyg.

[17] Bull PC, Lowe BS, Kortok M, Molyneux CS, Newbold CI, Marsh K (1998). Parasite antigens on the infected red cell surface are targets for naturally acquired immunity to malaria. Nat Med.

[18] Ofori MF, Dodoo D, Staalsoe T, Kurtzhals JAL, Koram K, Theander TG (2002). Malaria-induced acquisition of antibodies to *Plasmodium falciparum* variant surface antigens. Infect Immun.

[19] Kinyanjui SM, Bull P, Newbold CI, Marsh K (2003). Kinetics of antibody responses to *Plasmodium falciparum*-infected erythrocyte variant surface antigens. J Infect Dis.

[20] Chan J-A, Howell KB, Reiling L, Ataide R, Mackintosh CL, Fowkes FJI (2012). Targets of antibodies against *Plasmodium falciparum*-infected erythrocytes in malaria immunity. J Clin Invest..

[21] Baruch DI, Gamain B, Barnwell JW, Sullivan JS, Stowers A, Galland GG (2002). Immunization of *Aotus* monkeys with a functional domain of the *Plasmodium falciparum* variant antigen induces protection against a lethal parasite line. Proc Natl Acad Sci USA.

[22] Ghumra A, Khunrae P, Ataide R, Raza A, Rogerson SJ, Higgins MK (2011). Immunisation with recombinant PfEMP1 domains elicits functional rosette-inhibiting and phagocytosis-inducing antibodies to *Plasmodium falciparum*. PLoS ONE.

[23] Moll K, Kaneko A, Scherf A, Wahlgren M. Methods in malaria research. Sixth edition. MR4/ATCC Manassas; 2013. https://www.beiresources.org/portals/2/MR4/Methods_In_Malaria_Research-6th_edition.pdf.

[24] Angeletti D, Albrecht L, Wahlgren M, Moll K (2013). Analysis of antibody induction upon immunization with distinct NTS-DBL1α-domains of PfEMP1 from rosetting *Plasmodium falciparum* parasites. Malar J.

[25] Angeletti D, Albrecht L, Blomqvist K, Quintana MDP, Akhter T, Bächle SM (2012). *Plasmodium falciparum* Rosetting epitopes converge in the SD3-loop of PfEMP1-DBL1α. PLoS ONE.

[26] Nilsson S, Moll K, Angeletti D, Albrecht L, Kursula I, Jiang N (2011). Characterization of the Duffy-binding-like domain of *Plasmodium falciparum* blood-stage antigen 332. Malar Res Treat..

[27] Albrecht L, Moll K, Blomqvist K, Normark J, Chen Q, Wahlgren M (2011). *var* gene transcription and PfEMP1 expression in the rosetting and cytoadhesive *Plasmodium falciparum* clone FCR3S1.2. Malar J.

[28] Blomqvist K, Albrecht L, Quintana MDP, Angeletti D, Joannin N, Chêne A (2013). A sequence in subdomain 2 of DBL1α of *Plasmodium falciparum* erythrocyte membrane protein 1 induces strain transcending antibodies. PLoS ONE.

[29] Treutiger C-J, Hedlund I, Helmby H, Carlson J, Jepson A, Twumasi P (1992). Rosette formation in *Plasmodium falciparum* isolates and anti-rosette activity of sera from Gambians with cerebral or uncomplicated malaria. Am J Trop Med Hyg.

[30] Fleit HB, Kobasiuk CD (1991). The human monocyte-like cell line THP-1 expresses FcyRl and FcyRIl. J Leukoc Biol.

[31] Auwerx J, Staels B, Van Vaeck F, Ceuppens JL (1992). Changes in IgG Fc receptor expression induced by phorbol 12-myristate 13-acetate treatment of THP-1 monocytic leukemia cells. Leuk Res.

[32] Daigneault M, Preston JA, Marriott HM, Whyte MKB, Dockrell DH (2010). The identification of markers of macrophage differentiation in PMA-stimulated THP-1 cells and monocyte-derived macrophages. PLoS ONE.

[33] Unkeless JC, Scigliano E, Freedman VH (1988). Structure and function of human and murine receptors for IgG. Annu Rev Immunol.

[34] Micusan VV, Borduas AG (1977). Biological properties of goat immunoglobulins G. Immunology.

[35] Alexander EL, Sanders SK (1977). F(ab’)_2_ reagents are not required ig goat, rather than rabbit, antibodies are used to detect human surface immunoglobulin. J Immunol..

[36] Celada A, Cruchaud A, Perrin LH (1982). Opsonic activity of human immune serum on in vitro phagocytosis of *Plasmodium falciparum* infected red blood cells by monocytes. Clin Exp Immunol.

[37] Groux H, Gysin J (1990). Opsonization as an effector mechanism in human protection against asexual blood stages of *Plasmodium falciparum*: functional role of IgG subclasses. Res Immunol.

[38] Abdel-Latif MS, Khattab A, Kremsner PG, Klinkert M-Q, Lindenthal C (2002). Recognition of variant Rifin antigens by human antibodies induced during natural *Plasmodium falciparum* infections. Infect Immun.

[39] Abdel-Latif MS, Cabrera G, Köhler C, Kremsner PG, Luty AJF (2004). Antibodies to Rifin: a component of naturally acquired responses to *Plasmodium falciparum* variant surface antigens on infected erythrocytes. Am J Trop Med Hyg.

[40] Kaewthamasorn M, Yahata K, Alexandre JSF, Xangsayarath P, Nakazawa S, Torii M (2012). Stable allele frequency distribution of the polymorphic region of SURFIN(4.2) in *Plasmodium falciparum* isolates from Thailand. Parasitol Int.

[41] Osier FH, Feng G, Boyle MJ, Langer C, Zhou J, Richards JS (2014). Opsonic phagocytosis of *Plasmodium falciparum* merozoites: mechanism in human immunity and a correlate of protection against malaria. BMC Med.

