# Supplementary figures and images for: Phagocytosis-inducing antibodies to Plasmodium falciparum upon immunization with a recombinant PfEMP1 NTS-DBL1α domain
Source: Malar J. 2016 Aug 17;15:416. doi: 10.1186/s12936-016-1459-3 (PMC4987995; doi:10.1186/s12936-016-1459-3)

**Figure S2.**


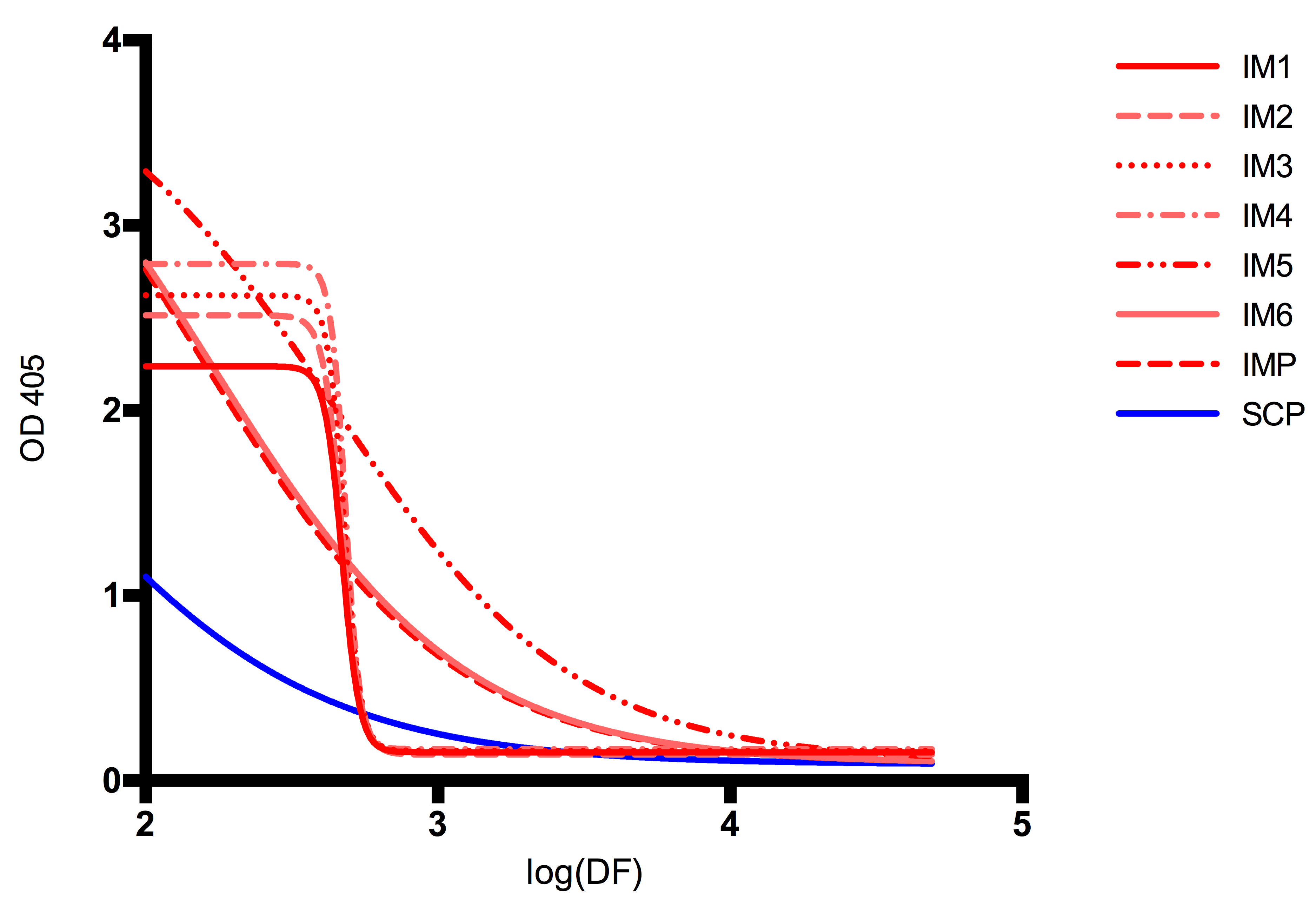

Supplement: Supplementary file 1 — 10.1186/s12936-016-1459-3 IgG levels in the human samples against the recombinant NTS-DBL1α (ITvar60) measured by ELISA. The original OD values were fitted to a 4 Parameter Logistic (4PL) curve. The antibody concentration is expressed as log of the dilution factor (DF). Immune samples are depicted in red and named from 1 to 6, The pooled immune sample (IMP) is also depicted. Swedish control pool (SCP) is depicted in blue. [file 12936_2016_1459_MOESM1_ESM.docx]
