# Supplementary material for: Phagocytosis-inducing antibodies to Plasmodium falciparum upon immunization with a recombinant PfEMP1 NTS-DBL1α domain
Source: Malar J. 2016 Aug 17;15:416. doi: 10.1186/s12936-016-1459-3 (PMC4987995; doi:10.1186/s12936-016-1459-3)
Supplement: Supplementary file 2 — 10.1186/s12936-016-1459-3 Surface reactivity of the human samples measured by FACS. Immune samples are depicted in red and named from 1 to 6, The pooled immune sample (IMP) is also depicted. Swedish control pool (SCP) is depicted in blue. [file 12936_2016_1459_MOESM2_ESM.docx]

1. IM1 **B.** IM2 **C.** IM3 **D.** IM4

**E.** IM5 **F.** IM6 **G.** IMP
